# Underwater Optical-Sonar Image Fusion Systems

**DOI:** 10.3390/s22218445

**Published:** 2022-11-03

**Authors:** Hong-Gi Kim, Jungmin Seo, Soo Mee Kim

**Affiliations:** 1 Ocean Science and Technology School, Korea Maritime and Ocean University, Busan 49112, Korea; 2 Maritime ICT R&D Center, Korea Institute of Ocean Science & Technology, Busan 49111, Korea

**Keywords:** underwater visualization, optical and sonar image fusion system, geometric calibration of multi-imaging systems, image fusion, single-image enhancement

## Abstract

Unmanned underwater operations using remotely operated vehicles or unmanned surface vehicles are increasing in recent times, and this guarantees human safety and work efficiency. Optical cameras and multi-beam sonars are generally used as imaging sensors in underwater environments. However, the obtained underwater images are difficult to understand intuitively, owing to noise and distortion. In this study, we developed an optical and sonar image fusion system that integrates the color and distance information from two different images. The enhanced optical and sonar images were fused using calibrated transformation matrices, and the underwater image quality measure (UIQM) and underwater color image quality evaluation (UCIQE) were used as metrics to evaluate the performance of the proposed system. Compared with the original underwater image, image fusion increased the mean UIQM and UCIQE by 94% and 27%, respectively. The contrast-to-noise ratio was increased six times after applying the median filter and gamma correction. The fused image in sonar image coordinates showed qualitatively good spatial agreement and the average IoU was 75% between the optical and sonar pixels in the fused images. The optical-sonar fusion system will help to visualize and understand well underwater situations with color and distance information for unmanned works.

## 1. Introduction

**Figure 1:**
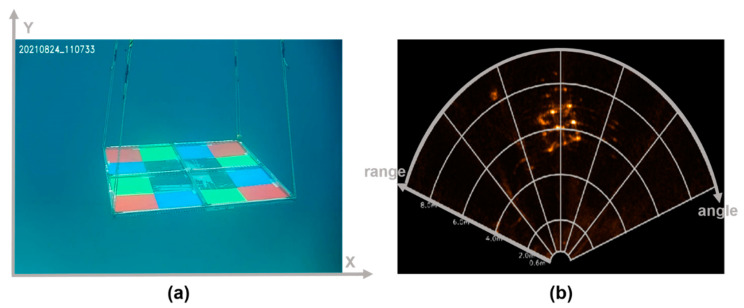
Two image coordinate systems, (**a**) Cartesian and (**b**) fan-shaped image coordinate systems of optical and multi-beam sonar sensors.

Recently, the need for unmanned vehicles, such as remotely operated vehicles and autonomous underwater vehicles, has increased due to the high safety and efficiency in tasks, such as surveying underwater structures and acquiring seabed data. For unmanned vehicles, imaging sensors are essential for visualizing underwater situations. Underwater optical cameras and multi-beam imaging sonars are the most commonly used imaging sensors. As shown in Figure 1, optical and sonar images are expressed in Cartesian and fan-shaped image coordinates, respectively. An optical camera provides an intuitive expression of underwater situations using red, green, and blue (RGB) color signals of the measured light signal. Since the light reaching the camera undergoes physical distortions, such as attenuation and reflection by water and floating particles, underwater optical images suffer from color casting and low visibility [1]. Multi-beam sonars provide images expressed in the fan-shaped image coordinate of the direction (angle, *θ*) and distance (range, *r*) calculated from the time-of-flight of the sonic beam. Sonic beams are well transmitted through water, but it is difficult to understand sonar images due to the low signal-to-noise ratio [2]. Several studies have been conducted to improve the quality of underwater optical and sonar images. Table 1 summarizes the single-enhancement techniques for optical and sonar images.

To compensate for wavelength-dependent color casting and low visibility, three representative approaches of single-image enhancement have been reported: conventional image processing-based, image formation model (IFM)-based, and deep learning-based enhancements. Conventional image processing algorithms, such as contrast-limited adaptive histogram equalization (CLAHE), homomorphic filtering, empirical mode decomposition, and multi-processing step-based techniques have been applied to underwater images for color compensation, histogram equalization, and boundary enhancement. However, these algorithms do not consider the spatial variance of the degradation over the field of view [3,4,5,6]. The IFM can be simplified as shown in Equation (1), where *I* is the measured light intensity of a pixel in the image, *J* is the restored light intensity, *t* is the light transmission map, and *A* is the background light:

(1) $$I\left(x\right)=J\left(x\right)t\left(x\right)+A\left(1-t\left(x\right)\right)$$

To restore J from I based on Equation (1), it is necessary to estimate the transmission map and background light relevant to the flight path of the measured light and the physical characteristics of the underwater environment. The dark channel prior (DCP) and gradient domain transform restore underwater images with physical prior knowledge of light, or through domain transform to estimate the transmission map [7,8,9]. IFM-based enhancement is effective in removing haziness in underwater images. However, there is a tradeoff between computational complexity and enhancement performance. Recently, deep learning methods, such as convolutional neural networks (CNNs) and generative adversarial networks (GANs) have been applied for transmission map estimation and white balancing [10,11,12]. The trained generator network of the underwater GAN and fast underwater image-enhancement GAN are enhanced from the underwater image to the cleaner image. Although uncertainty is minimized due to inaccurate prior knowledge and computational complexity of accurate IFMs, the performance of deep learning-based enhancement depends on robust construction of training datasets.

Single-image enhancement techniques for multi-beam sonar images have been proposed to remove noise and increase image contrast. Conventionally, the quality of sonar images is improved using filters or deep learning for noise removal and contrast enhancement. The median filter, which chooses a median value among the ascending-sorted pixel values in a kernel, is conventionally applied to remove random noise from the sonar image [13]. Gabor filters improve contrast and reduce noise in underwater sonar images [14]. A new adaptive cultural algorithm (NACA) optimized the filtering parameter for denoising sonar images [15]. In addition, CNNs are actively applied for noise reduction, crosstalk removal, and increasing the image resolution [16,17,18].

There have been some reports on fusion techniques for multiple imaging sensors. Two different datasets measured from acoustic and stereo cameras were fused by extrinsic calibration and feature matching [19,20]. The data measured from two 3D imaging sensors, an acoustic camera and stereo camera, were aligned and fused using point-to-point correspondence [19]. Opti-acoustic stereo imaging is performed by matching the structural features of the edges and specific points in the data measured from multiple sensors [20].

**Table 1:** Summary of single-image enhancement techniques for optical and multi-beam sonar images.

Image Type	Enhancement Method [Reference]	Description
Optical image	Empirical mode decomposition [3]	Decompose the color spectrum components of underwater images, and improve the images by applying different weights on the color spectrum components
	CLAHE-mix [4]	Apply CLAHE on the image in RGB and HSV color models and combine two contrast-enhanced images by Euclidean norm
	Image fusion [5]	Apply three successive steps of white balancing, contrast and edge enhancing, and fusing
	CLAHE-HF [6]	Enhance contrast of underwater images by CLAHE, and reduce noise by homomorphic filtering (HF)
	Red channel restoration model [7]	Apply a red channel model, which is a variation of DCP, to improve the most attenuated red channel signal of the underwater image
	Underwater IFM-based algorithm [8]	Recover the original image with the determined transmission map of direct transmitted, forward and backward scattered light
	DCP and depth transmission map [9]	Fuse DCP and depth map, which are the difference between the bright and the dark channels and the difference of wavelength-dependent light absorption, respectively
	UGAN [10]	Train underwater GAN (UGAN) from the paired clean and underwater images to learn the difference between the paired images, and generate enhanced underwater images using the trained UGAN
	CNNs for estimation of transmission and global ambient light [11]	Train two parallel CNN branches to estimate the blue channel transmission map and global ambient light signal
	FUnIE-GAN [12]	Train fast underwater image enhancement GAN (FUnIE-GAN) to learn global content, color, texture, and style information of underwater images
Sonar image	Median filter [13]	Reduce noise in sonar images by median filter
	Gabor filter [14]	Improve edge signal in sonar images by Gabor filter
	NACA [15]	Apply adaptive initialization algorithm to obtain a better initial clustering center and quantum inspired shuffled frog leaping algorithm to update cultural individuals
	CNN based auto encoder [16]	Train auto encoder from 13,650 multi-beam sonar images for enhancing resolution and denoising
	GAN based algorithm [17]	Train GAN using high- and low-resolution sonar image pairs for enhancing resolution
	YOLO [18]	Train you only look once (YOLO) network from the crosstalk noise sonar image dataset, and then remove the detected crosstalk noise

In this study, we developed an underwater optical-sonar fusion system that can simultaneously record optical and multi-beam sonar images, enhance both images, and then fuse the RGB color of the enhanced optical image and the distance of the enhanced sonar image. For optical image enhancement, we chose the image fusion method according to reports on the qualitative and quantitative comparisons of different single image enhancement techniques [21,22]. For sonar image enhancement, median filter and gamma correction method were applied to reduce noise and to enhance contrast because they are conventionally used on sonar images [23]. For image fusion, we performed geometric calibration with an RGB phantom, and estimated the transformation matrix between different optical and sonar image coordinates.

## 2. Materials and Methods

### 2.1. Underwater Optical-Sonar Fusion System

**Figure 2:**
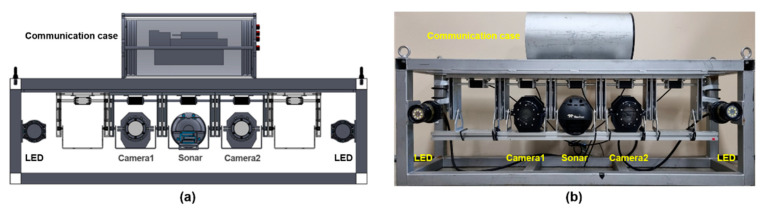
Optical-sonar fusion system, (**a**) schematic design and (**b**) real hardware comprising two underwater cameras and one multi-beam sonar.

**Figure 3:**
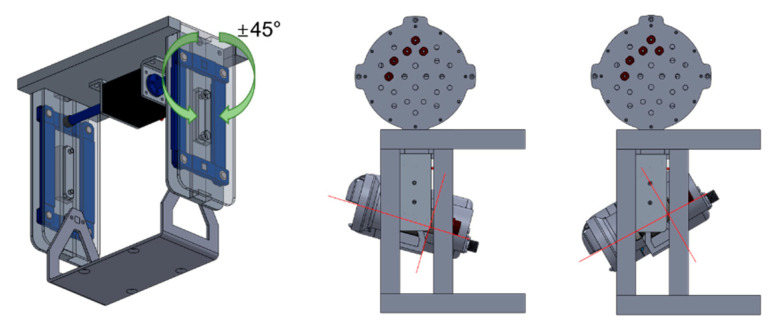
Schematic of the bracket equipped with a servo motor for tilting imaging sensors.

We developed an optical-sonar fusion system comprising two underwater cameras (Otaq, Eagle IPZ/4000, Lancaster, UK), a multi-beam sonar (Teledyne marine, Blueview M900-2250, Daytona Beach, FL, USA), two light-emitting diode (LED) lights (Deepsea power & light, LED SeaLite, San Diego, CA, USA), and a communication case, as shown in Figure 2. The communication box is an aluminum watertight case with power and data communication cables. Table 2 summarizes the specifications of the imaging sensors and LED lights. Each imaging sensor was attached to a movable bracket, which was equipped with a servo motor (Cehai Tech, D30, Qingdao, China) to control the tilting sensors. As shown in Figure 3, the bracket is designed for ±45° tilting with a servo motor operating at up to 30 kgf.cm torque and 270° angle. The optical-sonar fusion system weighs approximately 80 kg and 30 kg in air and water, respectively.

**Figure 4:**
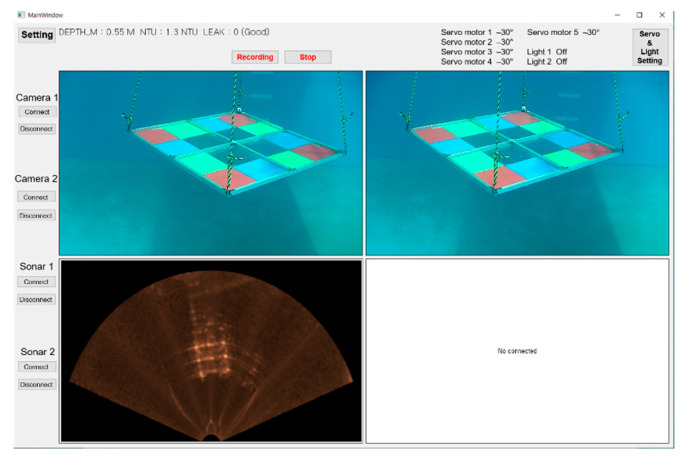
Software to control the display and acquisition of optical and sonar images, light operation, and enhancing and fusing both images.

Figure 4 shows a graphical user interface software supporting real-time visualization and simultaneous acquisition of optical and sonar images, turning on/off lights, and performing single-enhancement and optical-sonar fusion.

### 2.2. Enhancement of Underwater Optical and Sonar Images

In this study, we applied image fusion comprising three successive image processing steps: balancing, enhancing contrast and sharpness, and fusing two enhanced images to improve the color tone and sharpness of underwater optical images [5]. The first step, white balancing, compensates for the values of the red and green color channels of a pixel, $WB_{r}$ and $WB_{g}$ via Equations (2) and (3) to reduce the difference between the average RGB color channels ($\bar{I_{r}},\;\bar{I_{g}},\;\bar{I_{b}}$). The weights, α and  β, were experimentally determined in the range of 1.8 to 2.3 and 1.3 to 1.8, respectively:

(2) $$WB_{r}\left(x,y\right)=I_{r}\left(x,y\right)+\alpha \left({\bar{I}}_{g}-{\bar{I}}_{r}\right)\left(1-I_{r}\left(x,y\right)\right)\left(I_{g}\left(x,y\right)\right)$$

(3) $$WB_{b}\left(x,y\right)=I_{b}\left(x,y\right)+\beta \left({\bar{I}}_{g}-{\bar{I}}_{b}\right)\left(1-I_{b}\left(x,y\right)\right)\left(I_{g}\left(x,y\right)\right)$$

In the second step, CLAHE and the unsharp masking principle (UMP) were adopted to enhance the contrast and sharpness of the white-balanced image. CLAHE performs histogram equalization on the multiple sub-patches of an image and combines the equalized sub-patches. In Equation (4), the UMP sharpens the white-balanced image by weighted addition of the difference between the original and Gaussian filtered (G⊗WB) images:

(4) $$UMP\left\{WB\right\}=WB+\gamma \left(WB-\left(G⊗WB\right)\right)$$

Finally, the enhanced image ($\bar{J}$) was obtained by the weighted sum of the enhanced image by CLAHE and UMP in Equation (5). The weights $\omega_{1}$ and $\omega_{2}$ are determined using the normalized Laplacian contrast and saturation factors:

(5) $$\bar{J}=\omega_{1}CLAHE\left\{WB\right\}+\omega_{2}UMP\left\{WB\right\}$$

To reduce noise and enhance the contrast of multi-beam sonar images, we applied a median filter and gamma correction. The median filter selects the median value among the pixels in a 5 × 5 patch, and the center pixel in the patch is replaced by the median value. Consequently, values that are significantly higher or lower than the neighboring pixels in the patch can be removed. Gamma correction in Equation (6) corrects the input sonar pixel, $I\left(x,y\right)$ with a nonlinear weight ($\gamma_{2}$). In this study, we set $\gamma_{2}$ to 0.2:

(6) $$J\left(x,y\right)=255*{\left(\frac{I\left(x,y\right)}{255}\right)}^{\gamma_{2}}$$

### 2.3. Calibration and Fusion of Underwater Optical-Sonar Fusion System

**Figure 5:**
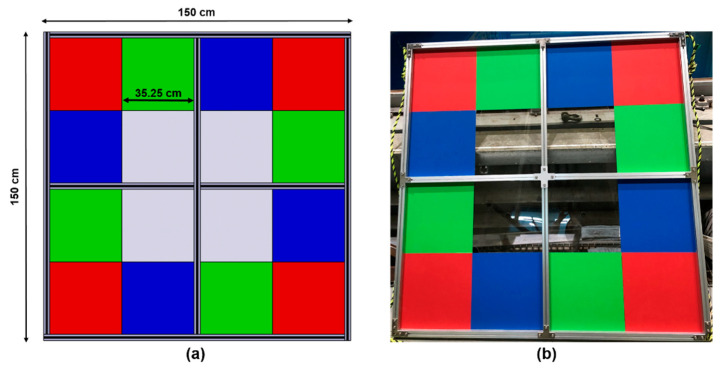
Calibration phantom, (**a**) schematic and (**b**) real RGB phantom for geometric calibration of underwater optical-sonar fusion system.

To fuse two different image coordinates, we first need to calibrate the two image sensors geometrically. For geometric calibration, we designed an RGB phantom that can be captured by optical and sonar image sensors, as shown in Figure 5. The size of the RGB phantom is 1.5 × 1.5 m in width and height, and the colored RGB aluminum plates and the transparent acrylic plates are aligned to construct various color and shape patterns. Each plate was 35.25 cm in width and height, and the weight of the RGB phantom was 40 kg in air.

**Figure 6:**
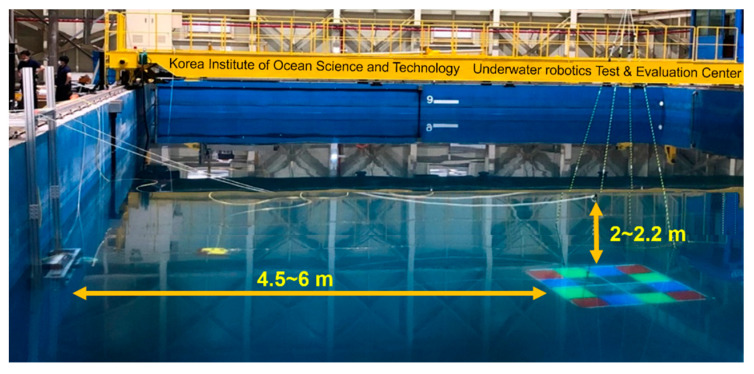
Experimental setup of the RGB phantom and optical-sonar fusion system for acquisition of calibration data.

Figure 6 shows the experimental setup in the water tank (Underwater test and evaluation center, Pohang, Korea). The water tank is 20 m in width, 35 m in height, and the maximum depth is 9.6 m. The optical-sonar fusion system was installed on the wall of water tank 0.5 m below water surface. We obtained simultaneous optical and sonar images of the RGB phantom. The phantom was located at a distance of 4.5 m and at a depth of 2 m. At a depth of 2.2 m from the water surface, the RGB phantom was moved at a distance of 5, 5.5, and 6 m from the image fusion system. At each distance and depth, the phantom was rotated by an angle of 15, 30, and 45 degrees on the left and the right. Table 3 summarizes 28 different locations of RGB phantom to acquire calibration data. By acquiring the calibration image data with the phantom placed at various locations in field of view, the calibrated transformation matrices could reflect the different geometric relationships between the corresponding pixels in sonar and optical image coordinates.

**Figure 7:**
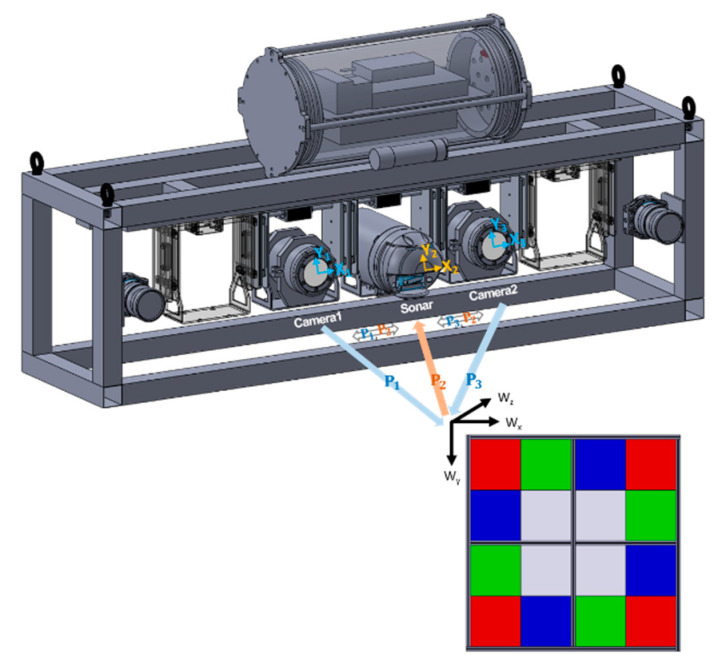
World coordinates ($W_{x},\;W_{y},W_{z}$), optical, and sonar image coordinates ($X_{n},\;Y_{n};n=1,2,3$).

Using calibration data, we estimated transformation matrices, $P_{n}\forall n=1,2,3$ between the world coordinate and two optical (n=1,3) and a sonar (n=2) image coordinate, as shown in Figure 7. Equation (7) represents the transformation from the world to image coordinates [24]:

(7) $$\left[\begin{array}{ccc} X_{n} & Y_{n} & 1 \end{array}\right]=\left[\begin{array}{cc} \begin{array}{cc} W_{x} & W_{y} \end{array} & \begin{array}{cc} W_{z} & 1 \end{array} \end{array}\right]\;\cdot \;P_{n},\;P_{n}=\left[\begin{array}{c} R_{n}\\ T_{n} \end{array}\right]\;\cdot K_{n}$$

Each transformation matrix $P_{n}$ in Equation (7) comprises an extrinsic parameter matrix and an intrinsic parameter matrix. The extrinsic matrix, which indicates the location of the optical camera and sonar in world coordinates, is represented by a 3×3 rotation ($R_{n}$) and a 1×3 translation ($T_{n}$) matrix. The 3×3 intrinsic matrix ($K_{n}$) expresses the characteristics of the image sensors, such as focal length, principal point, and skew coefficient, based on the pinhole camera model. Unlike optical images, sonar images are expressed in fan-shaped coordinate systems with the shooting angle of multi-sonic beams and the distance measured from the time-of-flight of the returned sonic beams. Thus, to transform the optical image coordinates to sonar image coordinates, we successively conducted three coordinate conversion steps: (i) from optical image to world coordinates, (ii) from world to sonar Cartesian coordinates (*θ*, *r*), (iii) from sonar Cartesian coordinates to sonar image fan-shaped coordinates ($P_{f}$), as in Equation (8):

(8) $$\left[\begin{array}{cc} \theta & r \end{array}\right]=\left[\begin{array}{ccc} X_{1} & Y_{1} & 1 \end{array}\right]\cdot \;P_{1}{}^{-1}\cdot \;P_{2}\cdot \;P_{f}$$

**Figure 8:**
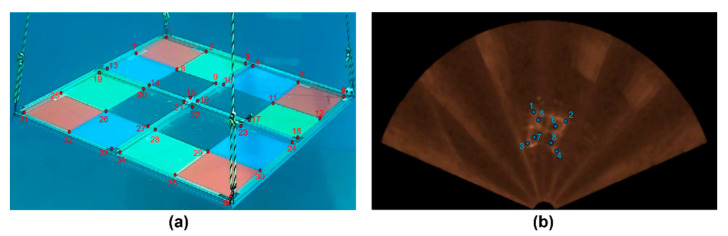
Corner detection numbering, (**a**) thirty-six optical and (**b**) eight sonar corner image points for estimating coordinate transformation matrices.

The acquired 28 calibration data of the simultaneous optical and sonar image pairs were used to estimate the transformation matrices $P_{n}$ in the Cartesian coordinate system. As shown in Figure 8, we manually extracted 36 and 8 corner image points from each simultaneously acquired optical and sonar image. The color difference of the plates in the RGB phantom creates corners in the optical images, and the material difference between the plates creates corners in sonar images.

## 3. Results

**Figure 9:**
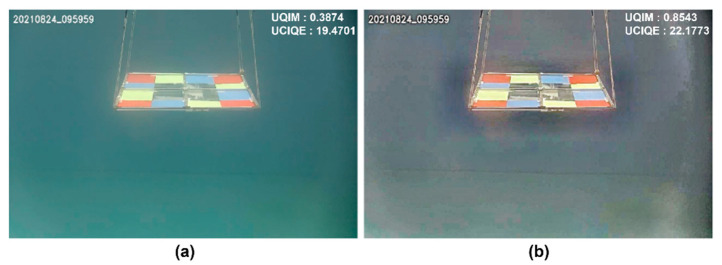
Single optical image enhancement, (**a**) original underwater optical image and (**b**) its enhanced image by image fusion.

Figure 9 qualitatively compares the original underwater optical image and the image enhanced by the image fusion technique. The greenish background in the original underwater image in Figure 9a was mitigated in the enhanced image (Figure 9b), and the overall color tone and edge signals corresponding to the RGB phantom were improved. In addition, the underwater image quality measure (UIQM) and underwater color image quality evaluation (UCIQE) were computed to quantitatively evaluate the optical color image quality [25,26]. In Equation (9), the UIQM is defined as the weighted sum of colorfulness (UICM), sharpness (UISM) and contrast (UIConM). UCIQE in Equation (10) is defined as the weighted sum of the chroma ($\sigma_{chr}$), luminance ($con_{Lum}$), and saturation ($\mu_{sat}$). The UIQM values are 0.39 and 0.85 before and after applying image fusion enhancement, respectively. UCIQE is evaluated as 19.47 and 22.18 before and after enhancement, respectively:

(9) UIQM=0.0282 UICM+0.298 UISM+0.034 UIConM

(10) $$UCIQE=0.468\;\sigma_{chr}+0.275\;con_{Lum}+0.258\;\mu_{sat}$$

The performance of image fusion was evaluated with 28 image pairs acquired from two cameras for calibration by calculating the mean and standard deviation (SD) of the UIQM and UCIQE. The mean and SD of UIQM and UCIQE of 56 underwater images were 0.56 ± 0.13 and 19.96 ± 1.15, respectively. After image fusion enhancement, the increased UIQM and UCIQE were 1.1 ± 0.17 and 25.53 ± 2.35, respectively. Compared with the original underwater image, image fusion increased the mean UIQM and UCIQE by 94% and 27%, respectively.

**Figure 10:**
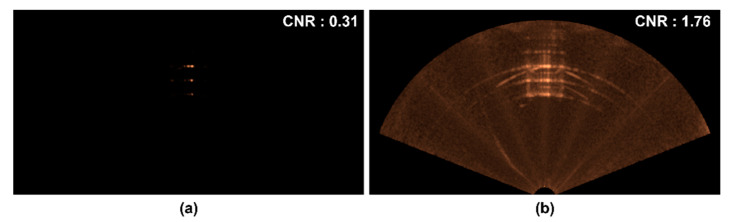
Single sonar image enhancement, (**a**) original sonar image and (**b**) its enhanced image by median filter (5 × 5) and gamma correction ($\gamma_{2}$ = 0.2).

Figure 10 shows the original sonar image and the enhanced image by median filter and gamma correction. The image after median filter and gamma correction showed more clear distinction of RGB phantom to the background than the original sonar image. In addition, we calculated the contrast-to-noise ratio (CNR) to evaluate the quality of sonar images using Equation (11) [27]. $S_{a}$ and $S_{b}$ are the average pixel values of region of interest (ROI) drawn on the background and on the RGB phantom regions of the sonar image, respectively. The noise term, σ, is the SD of the background ROI. The CNRs of before and after enhancement are 0.31 and 1.76, respectively. The CNR for 28 original sonar images was 0.33 ± 0.05 (mean ± SD), and the mean CNR was increased by six times after applying median filter and gamma correction (2.03 ± 0.12):

(11) $$CNR=\frac{\left|S_{a}\right.-\left.S_{b}\right|}{\sigma }$$

**Figure 11:**
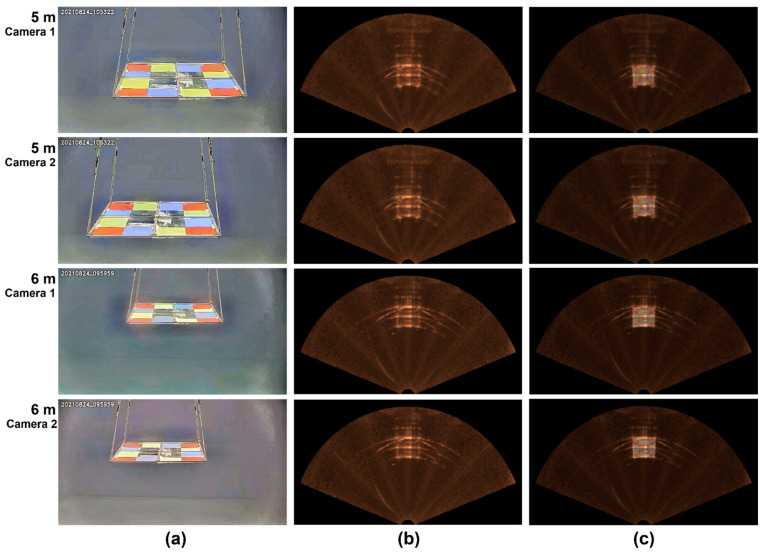
Enhanced and fused optical and sonar images taken at different distances of 5 and 6 m from the fusion system, (**a**) enhanced optical images (top and bottom rows are images measured from camera 1 and 2 at each distance, respectively), (**b**) enhanced sonar images, (**c**) overlayered optical color image on the sonar image.

Figure 11 shows the fused optical-sonar images with the calibrated transformation matrices after single-image enhancement. Both optical and sonar images were obtained simultaneously at a distance of 5 and 6 m from the optical-sonar fusion system. The fused image showed good spatial agreement between the optical and sonar images. The fused optical and sonar image in the fan-shaped coordinate provides simultaneously not only RGB color of the interested object, but also the distance. In order to evaluate quantitatively the spatial agreement between the optical and sonar images, Equation (12) calculates intersection over union (IoU). IoU is the intersected ($A_{o}$) to the union ($A_{u}$) area rate of the sonar and optical pixels corresponding to the RGB phantom region in the fused image. In Table 4, IoU at the distances of 5 and 6 m were 69 and 81% on average, respectively:

(12) $$IoU=\frac{A_{o}}{A_{u}}$$

## 4. Conclusions

We developed an optical-sonar fusion system with two underwater cameras and a multi-beam sonar and proposed a geometric calibration method for two different imaging sensors. In addition, we studied single-image enhancement techniques and multi-data fusion of optical cameras and multi-beam sonars, which are mainly used as imaging sensors for underwater tasks. To compensate for the color casting and low visibility of the optical images, we adopted image fusion comprising three steps: white balancing, contrast and edge enhancement, and enhanced image fusion. Noise reduction and contrast enhancement of the sonar images were conducted using a median filter and gamma correction. The single-enhancement techniques of optical and sonar images increased the visibility of the objects of interest in both images, and the figure of merit for performance evaluation was higher than that of the original underwater images. Both the enhanced optical and sonar images were fused with calibrated transformation matrices between different imaging coordinates. The fused image in sonar image coordinates showed qualitatively good spatial agreement and the average IoU was 75% between the optical and sonar pixels in the fused images. The calibration and fusion methods proposed in this study can be applied to other sonar systems, such as synthetic aperture sonar, by estimating the transformation matrix between two image coordinates from the paired corner points extracted from the simultaneously measured sonar and optical images [28,29]. Single-image enhancement techniques and the optical-sonar fusion system will help to visualize and understand underwater situations with color and distance information for unmanned works.

## Figures and Tables

**Table 2:** Specifications of two imaging sensors and the light in the optical-sonar fusion system.

Device	Specifications		
Eagle IPZ/4000	Field of view	3.3~45°	
	Spatial resolution	1920 × 1080	
Blueview M900-2250	Dual frequencies	900 kHz	2250 kHz
	Maximum range	100 m	10 m
	Field of view	130° (H) × 20° (V)	
LED SeaLite	Output	10,000 Lumens	
	Efficacy	63 lm/W	

**Table 3:** Different setup locations of the RGB phantom for acquisition of geometric calibration data.

Depth (m)	Distance between System and Phantom (m)	Rotation (°)
2	4.5	0, −15, −30, −45, 15, 30, 45
2.2	5, 5.5, 6	

**Table 4:** IoU result value by distance and camera.

Distance	Camera 1	Camera 2
5 m	67.7%	70.4%
6 m	77.1%	84.1%

## Data Availability

Not applicable.
